# Links of Previous Incarceration With Geriatric Syndromes and Chronic Health Conditions Among Older Adults in the US

**DOI:** 10.1093/geroni/igaf122.1354

**Published:** 2025-12-31

**Authors:** Christopher Kaufmann, Alexander Testa, Dylan Jackson, Meghan Novisky, Carmen Gutierrez, Jack Tsai, Adam Spira, Roland Thorpe

**Affiliations:** University of Florida, Gainesville, Florida, United States; UTHealth Houston, Houston, Texas, United States; Johns Hopkins University, Baltimore, Maryland, United States; Cleveland State University, Cleveland, Ohio, United States; The University of North Carolina at Chapel Hill, Chapel Hill, North Carolina, United States; UTHealth Houston, Houston, Texas, United States; Johns Hopkins Bloomberg School of Public Health, Baltimore, Maryland, United States; Johns Hopkins Bloomberg School of Public Health, Baltimore, Maryland, United States

## Abstract

This study investigated the association between previous incarceration and various geriatric and chronic health conditions among adults 50 and older in the United States. Data came from the National Longitudinal Study of Adolescent to Adult Health—Parent Study (AHPS) collected in 2015–2017, including 2,007 individuals who participated in the parent study (Parent Sample) and 976 individuals who participated in the spouse/partner study (Spouse/Partner Sample). Multiple logistic regression was used to investigate the relationship between previous incarceration and geriatric syndromes (dementia, difficulty walking, difficulty seeing, difficulty with activities of daily living) and chronic health conditions (self-reported poor/fair health, diagnosis of cancer, hypertension, diabetes, heart disease, stroke, chronic lung disease, depression, and alcohol use [4 or more drinks per week]). In adjusted analyses, respondents with previous incarceration in the AHPS had significantly higher odds of reporting difficulty walking, activities of daily living difficulty, cancer diagnosis, depression diagnosis, and chronic lung disease (adjusted odds ratios [aORs] = 2.21–2.95). Respondents in the AHPS spouse/partner study reported higher odds of difficulty seeing, cancer, depression, chronic lung disease, and heavy alcohol use (aORs = 1.02–2.15). Previous incarceration may have an adverse impact on healthy aging. Findings highlight the importance of addressing the enduring health impacts of incarceration, particularly as individuals transition into older adulthood.

